# The Importance of Postural Cues for Determining Eye Height in Immersive Virtual Reality

**DOI:** 10.1371/journal.pone.0127000

**Published:** 2015-05-18

**Authors:** Markus Leyrer, Sally A. Linkenauger, Heinrich H. Bülthoff, Betty J. Mohler

**Affiliations:** 1 Max Planck Institute for Biological Cybernetics, Tübingen, Germany; 2 Department of Psychology, Lancaster University, Lancaster, United Kingdom; 3 Department of Brain and Cognitive Engineering, Korea University, Seoul, South Korea; VU University Amsterdam, NETHERLANDS

## Abstract

In human perception, the ability to determine eye height is essential, because eye height is used to scale heights of objects, velocities, affordances and distances, all of which allow for successful environmental interaction. It is well understood that eye height is fundamental to determine many of these percepts. Yet, how eye height itself is provided is still largely unknown. While the information potentially specifying eye height in the real world is naturally coincident in an environment with a regular ground surface, these sources of information can be easily divergent in similar and common virtual reality scenarios. Thus, we conducted virtual reality experiments where we manipulated the virtual eye height in a distance perception task to investigate how eye height might be determined in such a scenario. We found that humans rely more on their postural cues for determining their eye height if there is a conflict between visual and postural information and little opportunity for perceptual-motor calibration is provided. This is demonstrated by the predictable variations in their distance estimates. Our results suggest that the eye height in such circumstances is informed by postural cues when estimating egocentric distances in virtual reality and consequently, does not depend on an internalized value for eye height.

## Introduction

Eye height is a reliable metric to scale for example the heights of objects [1, 2], velocities [3], affordances [4] and egocentric distances [5, 6]. All of these percepts are fundamental for successful interaction with our surrounding environment, whether real or virtual. The notion is that the distances to and heights of viewed objects are understood by using one’s eye height (i.e., the distance from the eyes to the ground) as a unit of measure with which to scale the world. The use of this term is in contrast to the concept of eye level, which could be considered mutable and more akin to line of sight. Eye level is important for perceiving the relationship between one’s eye height and sizes or distances (i.e. horizon ratio, see [6]), whereas eye height defines the unit of the scale [7]. In order to derive size and distance in terms of a metric, eye height must be known (alternatively, interpupillary distance could be used for scaling near-space distances); whereas, for determining object height relative to the body (i.e. the object is smaller than me), only eye level is required.

However, our eye height can change quite drastically during our everyday lives or while exploring a virtual environment. For example, during the course of a day, we constantly change our posture, and consequently our eye height changes accordingly. One may stand, sit down, stand again and then even recline, yet the bookshelf across the room is still perceived to be at the same distance and to have the same size most of the time. Thus, to be a useful metric, the known eye height needs to be flexible to ensure that the perception of the surrounding visual environment does not change due to such natural variations in eye height. How is the flexibility of this important metric ensured? If there is only little possibility for calibration (see [8], why calibration might be important), eye height could either be informed by experience, i.e. by a stable internalized value for eye height across different postures, or be informed by different sources of information in real-time.

In principle, there are multiple sources of information, which could be used to inform eye height in real-time to ensure the necessary flexibility throughout the naturally occurring variations of our eye height. However, theoretically we can distinguish between two main sources of sensory information specifying eye height: *visual* and *postural* sources. The former is composed of optical information like accommodation, convergence, binocular disparity and motion parallax and the latter is composed of proprioceptive and vestibular information [9]. When acting in a flat environment in the real world, both sources of information are naturally coincident. If they are not, recent research in the real world suggests that for example the eye height above a table surface can be informed by stereo vision, but only if one can calibrate to this visual information by performing a goal directed action over time [8, 10]. Thus, an experienced perception-action coupling might enable us to calibrate our eye height across different circumstances.

That such a calibration to new visual information may be necessary might be explained by the reliability of the visual information alone for determining eye height. As Warren & Whang [11] described, “the potential visual information itself like accommodation and convergence are ineffective for greater distances (e.g. beyond one meter) and binocular disparity does not provide absolute distance information” [11]. Furthermore, motion parallax, similar to accommodation and convergence, yields poor estimates of distance. However, not only calibration to potentially noisy visual information might help to determine eye height. Warren & Whang hypothesized that the body might also contribute important information for determining our eye height [11]. However, despite the findings that calibration might be necessary to inform eye height appropriately using stereo vision for a near space task [8, 10], empirical studies investigating how we might determine eye height for tasks in action space, especially if no calibration is allowed, are rare (to some extent [11, 12, 13]).

In the real world, the potential sensory information for determining eye height is naturally coincident most of the time (e.g. when acting on planar surfaces), whereas this information is easily dissociated in virtual environments. In addition, there is often little opportunity to give the user enough experience in virtual environments for calibration by providing them with the possibility to perform perception-action couplings for the given space, like reaching to objects [14] or walking around in the virtual world [15]. In contrast to the real world, where an experimental apparatus such as an adjustable surface where the height of the surface is unknown to the observer is needed, visual and postural information are easily decoupled (either by accident or on purpose) in a virtual reality scenario. Thus, we were interested in investigating how eye height in such a virtual reality scenario is determined, which in combination with egocentric distance perception might have different consequences on the perceived distance.

**Fig 1:**
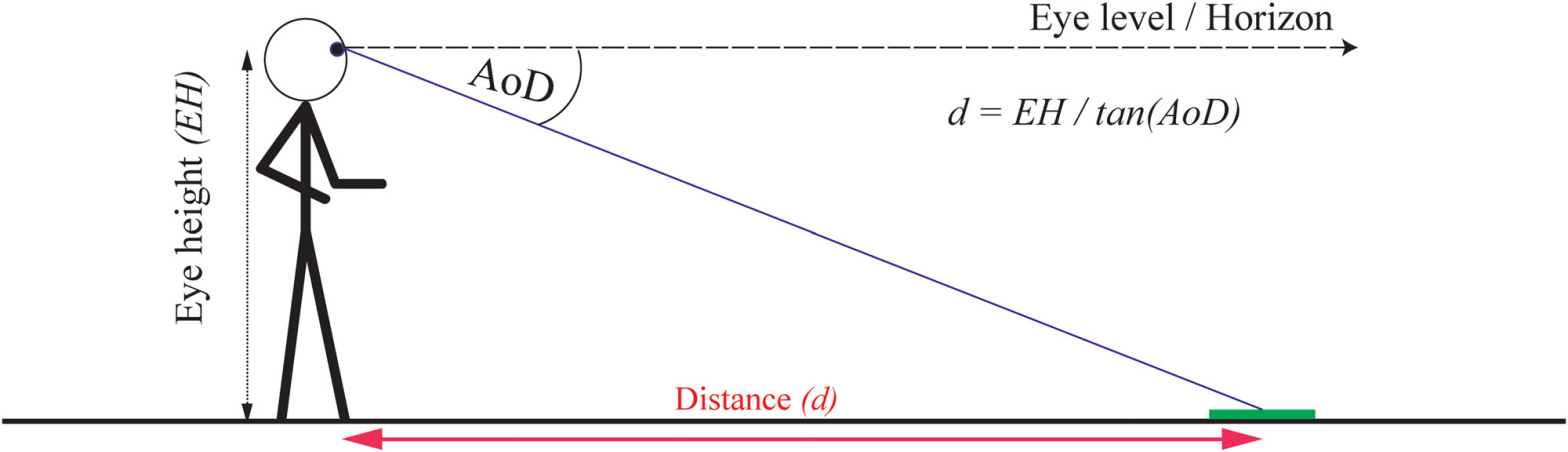
Egocentric distance perception using eye height and the angle of declination below the horizon to an object on the ground.

The angle of declination hypothesis [5, 6] states that the eye height of the observer and the visual angle to the target (angle of declination below the horizon) is used to determine distances to objects with physical contact to the ground (Fig 1). Sedgwick [6] proposed that the angle of declination below the horizon (AoD) to a target on the ground in combination with the (known) eye height (EH) of the observer can be used to determine distances (d) following the equation d = EH/tan(AoD) (see also [5, 16]). This tight coupling of eye height and perceived egocentric distances enables us to make predictions about how each potential source of information for determining eye height in VR portrayed in a head-mounted display (HMD) may influence perceived distance.

**Fig 2:**
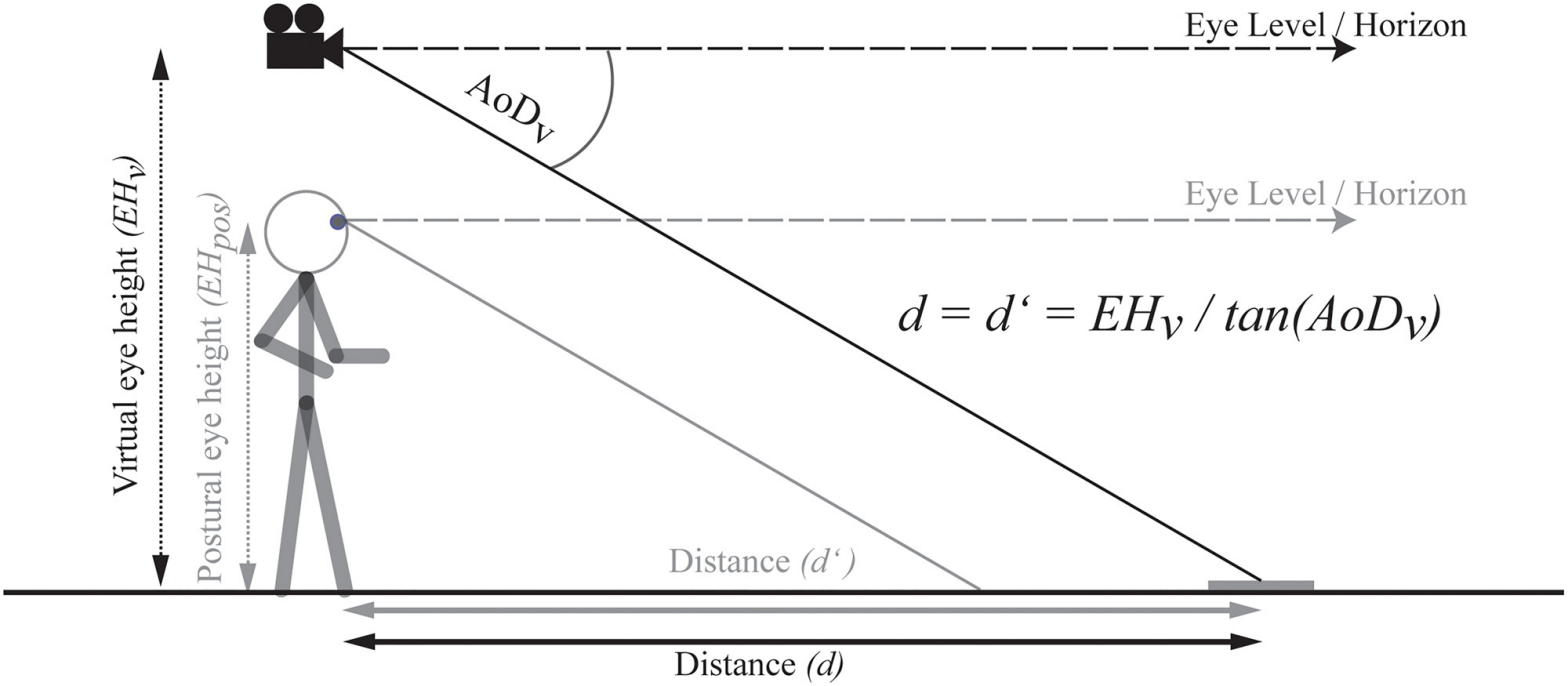
If eye height is informed by vision (EH_v_) and continuously informed by visual information across various environmental contexts, the ratio between eye height and tangent of the angle of declination (AoD_v_) and therefore the distance remains the same. **Note:** The camera symbol represents the manipulated virtual eye height in the VE.

**Fig 3:**
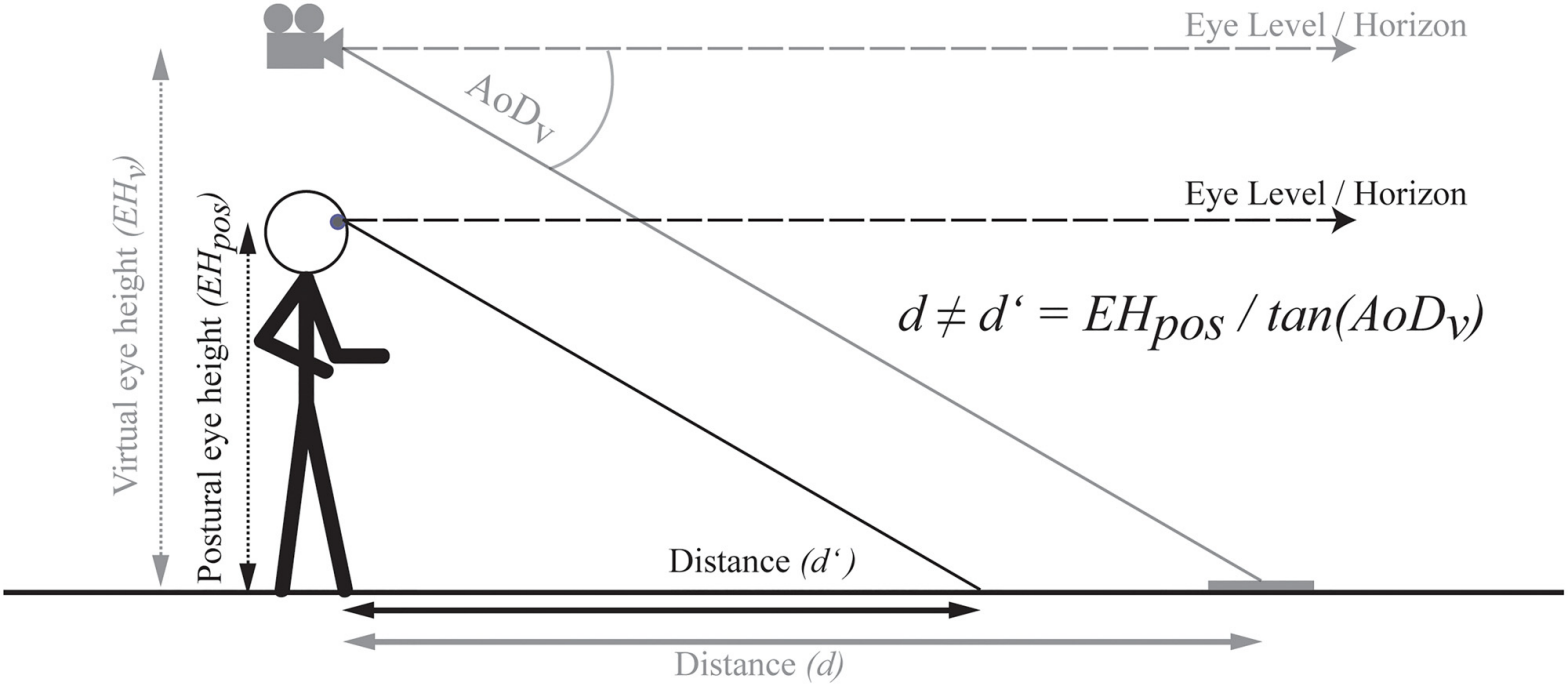
Prediction based on the use of a combination of the visually specified angle of declination (AoD_v_) and the postural eye height (EH_pos_) of the observer regardless of the potentially displayed environment. In the case illustrated, underestimation of the distance is predicted, whereas for a lowered virtual EH overestimation of the distance is predicted. **Note:** The camera symbol represents the manipulated virtual EH.

Specifically, suppose that observers assess their eye height by relying solely on visual information of the virtual surrounding environment displayed in the HMD. If this were true, then observers should determine their eye height depending on the visual information specifying depth to the ground surface and this should vary depending on e.g. their posture or manipulations to the visual information (i.e. their virtual eye height). In other words, any discrepancy between postural and visual cues in the specification of eye height should be negligible and the ratio between eye height and the angle of declination remains invariant. In such a case, perceived distance would be constant even when visually specified eye height in VR and the corresponding angle of declination change (Fig 2). Alternatively, if there is a discrepancy between the virtual and postural eye height and eye height is determined via postural cues (see Fig 3), the related visual angle from the different experienced virtual eye height should be combined with the postural eye height. Consequently, the angle of declination to the target changes with an increase in the virtual eye height. In combination with an unchanged postural eye height, an increase in the virtual eye height should result in decreases in perceived distance; whereas decreases in the virtual eye height should result in increases in perceived distance (see Fig 3).

However, because of visual limitations, specifically limitations in providing naturally occurring stereo cues such as matching accommodation and convergence cues when using 3D displays (see [14]), the visual information might not be reliable enough to be used for determining eye height without any possibility for calibration. Thus, postural cues may become more important as compared to a real world setting. For example, recent research has demonstrated that even when the observers were allowed to walk across a virtual room (and potentially calibrate), they ignore visual stereo cues in favour of a fictional stable world [17]. Consequently, the observers made large errors in size judgments after the virtual environment was dynamically altered in size. Thus, the participants might have been relying more on their unaltered postural information than what they visually perceived. Therefore, we hypothesize that our observers might rely more on their postural information for determining their eye height. Consequently, we expect that observers in a virtual environment determine their eye height by relying on postural cues, because they are not able to use or ignore visual information potentially indicating an altered eye height in a virtual environment, which should have predictable consequences on perceived egocentric distances in virtual environments.

Furthermore, we hypothesize that if eye height in virtual worlds is determined by postural cues, such a system is flexible enough to allow for a consistent perception of distance. For example when the observer is changing postures from standing to sitting or even lying in a bed, distance should be perceived as the same (similar to the perceived size of objects, see [1, 2]). Thus, the eye height used to determine distances should change according to changes in the posture of the body. To achieve such a constant perception of the virtual world the eye height unit could be internalized as a remembered eye height, as suggested by Sinai et al. [13]. Perceivers have exhaustive experience with various postures and the perceptual system could learn the relationship between the different eye heights of such postures over time. However, we expect that to assure perceptual constancy across various postures, eye height is determined with respect to changes in the body posture in real-time, which would eliminate the need for a stored representation of eye height based on prior experience.

To investigate our hypotheses, we conducted three experiments using a distance perception task in virtual environments, while manipulating the virtual (visual) eye height across different postures (Experiments 1–3). If our participants rely more on postural information in these circumstances, we expect predictable variations in the distance estimates following a manipulation of the virtual eye height. Furthermore, we conducted two experiments to investigate whether humans would be able to use the visual information present in virtual environments to determine their eye height or whether the visual information is unreliable due to known limitations of the VR technology.

## Experiment 1: Determining eye height in a standing posture in VR

Postural and the potentially visually specified (virtual) eye height were decoupled using VR. Participants experienced a visually taller or shorter eye height while standing on a flat (real and virtual) ground plane and estimated distances to targets, while they were not told that that the floor they saw in the VR scene was at the same height as the height of the physical floor they felt under their feet in the real world. Changes in the virtual eye height should be coupled with the corresponding changes in the angle of declination. As a result, if perceived eye height would be specified by using the available visual information, distance estimates should remain constant across different eye heights. However, if eye height in such circumstances is determined by postural cues, then increases and decreases in the virtual eye height should only influence the angle of declination with respect to determining distance. Thus, this change should lead to a compression of distances following an increase of visual eye height and expansion of distances following decreases in visual eye height compared to the baseline estimates where the virtual eye height is matching the postural eye height.

### Method

#### Participants

Fifty-four paid (26 female) participants were recruited from the university community of Tübingen, Germany. All had normal or corrected to normal visual acuity and could fuse stereo displays. The age ranged from 18 to 64 years (*M* = 29.13).

#### Ethics Statement

In this and all subsequent experiments, participants started by completing a written consent form. All experiments were performed in accordance with the 1964 Declaration of Helsinki and were approved by the ethical committee of the University of Tübingen. All participants were debriefed and informed of the purpose of the study at the end of the experiments. Individuals depicted in this manuscript (i.e. in images) have given written informed consent (as outlined in PLOS consent form) to publish these case details.

#### Stimuli and apparatus

We displayed a virtual environment consisting of a flat ground plane without any familiar size cues through an Nvis nVisor SX60 head-mounted display (Nvis Inc., Reston, VA, USA) with a resolution of 1280 × 1024 pixels per eye (in stereo). The head-mounted display (HMD) has a refresh rate of 60 Hz per eye and a contrast of 100:1. The field of view of the HMD is 60° diagonal, with a spatial resolution of approximately 2.2 arc-minutes per pixel. The HMD has collimated optics with a focal point at infinity, creating a virtual image, which appears to be at infinity rather than just a few centimeters from the face. This means that with the parallel display setup in the HMD the eyes converge and accommodate towards a virtual plane at simulated infinity. The position and orientation of the HMD was tracked by a 16-camera Vicon MX13 (Vicon, Oxford, UK) tracking system. The environment included a visual horizon and a blue sky. To provide a correct visual horizon, a software correction was implemented to compensate for radial distortion due to the optics of the HMD (if uncorrected, the horizon would appear as a curve at the edges of the optics). The ground plane in the virtual environment was textured with a random stone pattern to eliminate familiar size cues while still providing linear perspective cues (through tiling) and texture gradient cues. An octagonal green disc with a radius of 21.5 cm and a height of 1.4 cm indicated the distances to be judged.

#### Design and procedure

**Fig 4:**
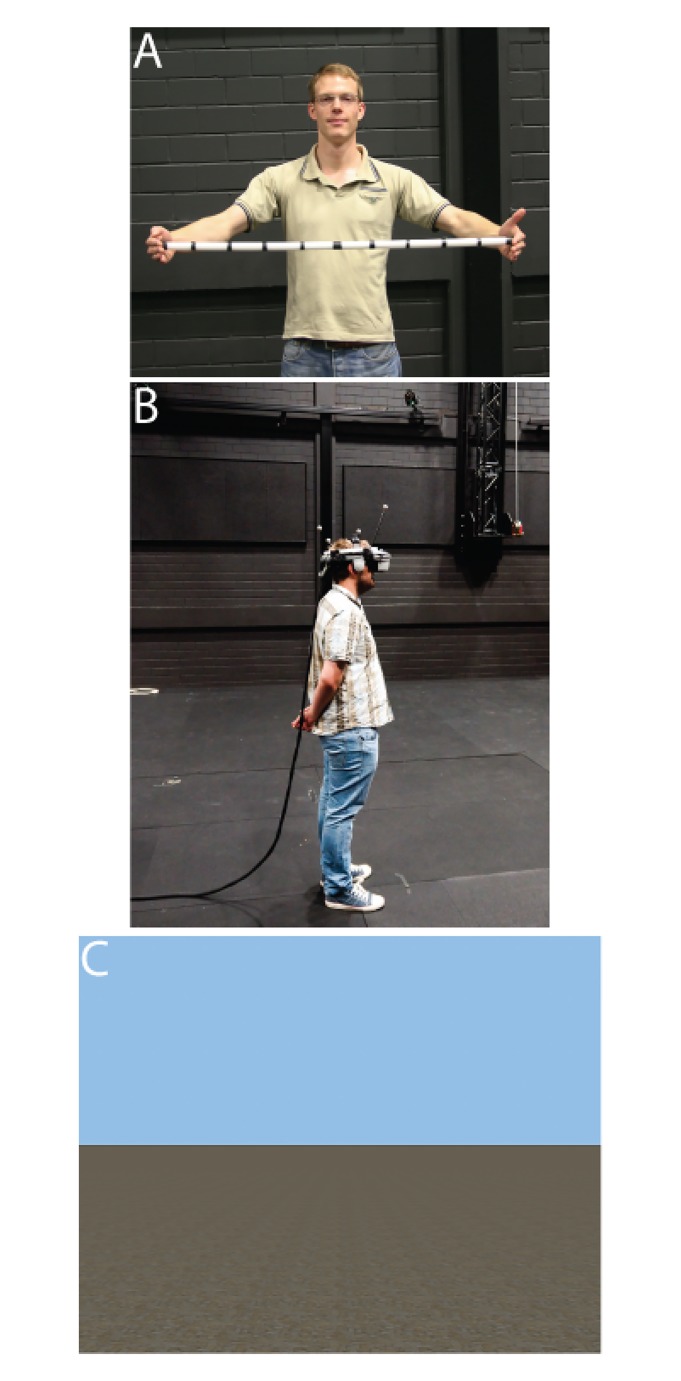
The used experimental setup. **A:** Experimenter showing the participant a meter stick with additional labels every 10 centimeters. **B:** Participant during the distance judgment task using the NVisor SX 60 HMD. **C:** The sparse-cue virtual environment used for Experiments 1–3. **Note:** The individuals in these images have given written informed consent (as outlined in PLOS consent form) to publish these case details.

All participants received written and verbal instructions and were shown a meter stick with additional labels every 10 centimeters, until they indicated that they had a good representation of the length of the stick. The participants were randomly assigned to only one of three conditions (between-subject design), in which the virtual eye height: (1) matched the postural eye height (0 cm), (2) was 50 cm lower than the postural eye height (-50 cm), or (3) was 50 cm higher than the postural eye height (+50 cm). The participants stood comfortably upright. They were not allowed to turn, bend, or lean forward or to the sides, nor were they allowed to deviate from their standing position (see Fig 4). In addition, in this and all subsequent experiments, the participants were not provided with (perceptual-motor) feedback from forward, backward or sideways locomotion. This allowed us to investigate, whether a change in visual or postural cues specifying eye height in virtual environments influences distance estimates in isolation and without the opportunity to calibrate actions to the visual cues by providing feedback about the target distance (c.f. [15]). However, because we used head tracking (position and orientation), motion parallax as a visual cue to eye height was available for all participants.

The experiment started with a 5 minute training phase to familiarize the participants with the virtual environment and give them the possibility to explore the environment (stationary—free head movements were allowed) and the manipulated eye height without any targets displayed. During this and the judgment phase all participants had the possibility to look down and were encouraged to do so. After the exploration phase, the target was displayed in the same environment at a certain distance, and the participants had as much time as they needed to judge the distance. When the participants indicated they were ready, the screen of the HMD was blanked; participants closed their eyes and turned their head 90° to the left and verbally reported the distance. Participants were instructed to report as accurately as they could in meters and centimeters. After reporting, the participants turned their head back, and following an indication of readiness from the participants, the next target was displayed. Participants completed 18 trials (4, 5 and 6 meters, each six times in a random blocked order).

### Results

We analyzed the verbal distance estimates using a repeated measures analysis of variance (ANOVA) with distance (4, 5, 6 m) and repetition (1–6) as within-subjects factors, virtual eye height (-50, 0, or +50 cm) as a between-subjects factor, and distance estimates as the dependent measure. As expected, distance was significant, with the estimates of distance increasing linearly from the 4 to 5 to 6 m distances, *F*(2, 102) = 382.88, *p* < .001, *ŋ*_*p*_^2^ = .88. Overall distances were compressed, which is a well-documented phenomenon in VR using HMDs (see for example [18, 19]).

**Fig 5:**
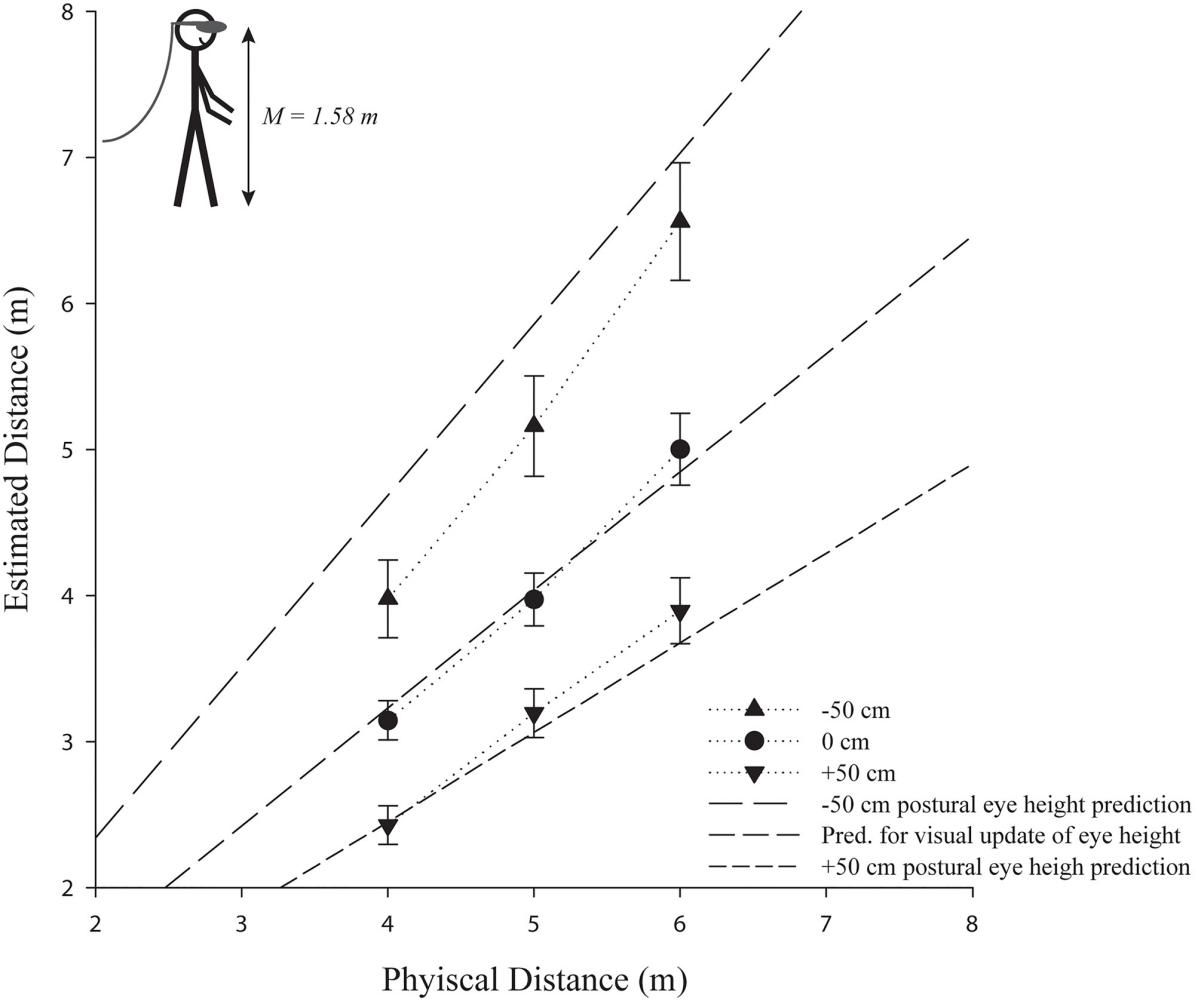
The effect of a manipulated virtual eye height (-50 cm or +50 cm) on egocentric distances in a standing position in comparison to the respective baseline condition (0 cm). Error bars represent ±1 SE. The actual mean participant (postural) eye height in the experiment is depicted in the left upper corner. **Note:** (a) The predictions are shifted by the observed underestimation in the baseline condition to account for the usually observed distance underestimation in head mounted displays (in an ideal world, the 0 cm estimates would correspond to veridical performance). (b) If the virtual eye height were used, there should be no differences and the prediction for visual eye height would apply for all conditions.

The repeated measures ANOVA also revealed that the eye height manipulation had a significant effect on the estimated distances in the -50 cm (*M* = 5.23, *SE* = 0.33), 0 cm (*M* = 4.04, *SE* = 0.18), and +50 cm (*M* = 3.17, *SE* = 0.17) eye height conditions, *F*(2, 51) = 18.85, *p* < .001, *ŋ*_*p*_^2^ = .43. This suggests that in this experiment postural eye height rather than the virtual eye height is used to determine the egocentric distances if these sources of information are in conflict and there is little possibility for calibration (see Fig 5). Post-hoc pairwise comparisons using Bonferroni correction confirmed significant differences between the -50 and 0 cm eye height conditions, *p* = .003, the 0 and +50 cm conditions, *p* = .039, and the -50 and +50 cm conditions, *p* < .001. In addition, there was an interaction between eye height condition and distance, *F*(4,204) = 10.84, *p* < .001, *ŋ*_*p*_^2^ = .30, with the differences between the eye height conditions increasing as a function of increase in distance, which is predicted by the postural eye height hypothesis (see Fig 3).

## Experiment 2: Determining eye height in a sitting posture

The results of Experiment 1 suggest that postural eye height is used to determine egocentric distances in a standing posture, if the information potentially specifying eye height in a virtual environment is in conflict. However, eye height needs to be flexible across various postures to achieve perceptual constancy (see e.g. [1]). We conducted another experiment using a standard sitting posture, which resulted in an approximately 50 cm shorter postural eye height compared to the standing eye height in Experiment 1. If eye height would be determined by postural cues changing with the posture of the body, we would expect estimates in the 0 cm condition to be comparable to those in Experiment 1 (0 cm) and underestimation to occur in the raised (+50 cm) condition.

### Method

#### Participants

Twenty-five paid (17 female) participants were recruited from the university community of Tübingen, Germany. All had normal or corrected to normal visual acuity and could fuse stereo images. Age ranged from 18 to 54 years (*M* = 29.08).

#### Stimuli and apparatus

The same technical setup and virtual environment as in Experiment 1 were used. In this experiment, the participants sat on a standard chair with 44 cm sitting height (with a 44 cm long × 46 cm wide seat). The chair was positioned on the ground at the same location on the floor where the participants were standing in Experiment 1.

#### Design and procedure

**Fig 6:**
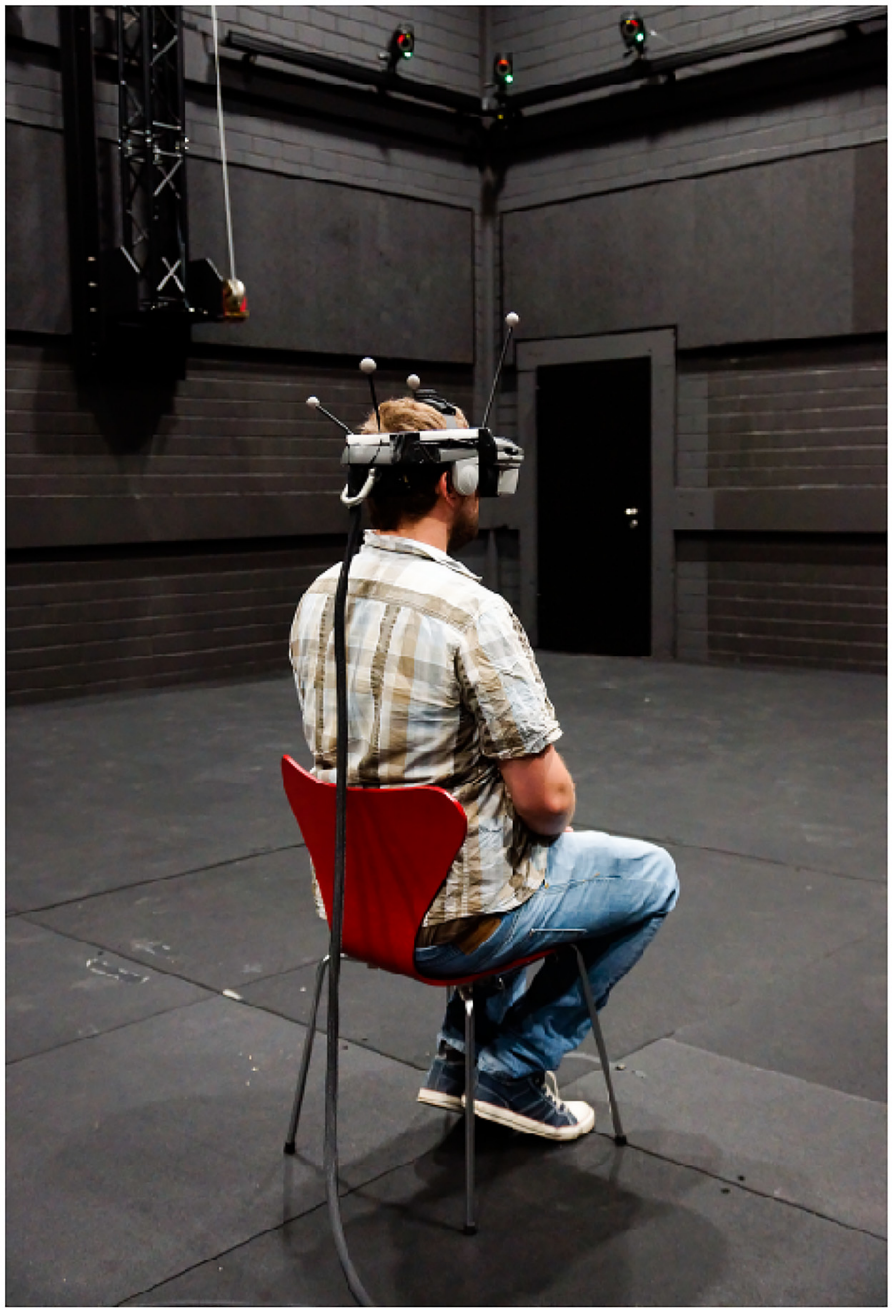
Participant judging distances in the sparse-cue virtual environment in a sitting posture. The individual in this image has given written informed consent (as outlined in PLOS consent form) to publish these case details.

The procedure was the same as in Experiment 1, except that participants were seated (see Fig 6). The participants were randomly assigned to only one of two conditions (between-subject design), either (1) a baseline condition where the visually specified eye height matched the actual seated eye height (0 cm) or (2) a 50 cm raised (+50 cm) visually specified eye height. We omitted the -50cm condition for this experiment as the -50 condition situated individuals in a pilot study so close to the ground plane that, given a seated posture and a moving head (with varying eye height), the virtual eye height that was not always positive (as in above the floor). Participants were instructed to sit upright and not to bend at the waist or lean forward to obtain a better view of the target. The participants were allowed to rotate their heads freely. The participants did not receive any feedback about the accuracy of their estimates. Participants completed 18 trials (4, 5 and 6 meters, each six times in a random blocked order). The procedure for reporting the distances was the same as in Experiment 1.

### Results

Two participants were removed from the analysis, one for being more than 3 *SD* above the mean and another after her admission of being a specialist in this research area. Distance estimates were analyzed using a repeated measures ANOVA with distance (4, 5, 6 m) and repetition (1–6) as within-subjects factors, virtual eye height (0 and +50 cm) as a between-subjects factor, and distance estimates as the dependent variable. As expected, distance was significant, with distance estimates increasing linearly with increasing distance, *F*(2, 42) = 371.68, *p* < .001, *ŋ*_*p*_^2^ = .95. We observed a similar distance compression as in Experiment 1.

**Fig 7:**
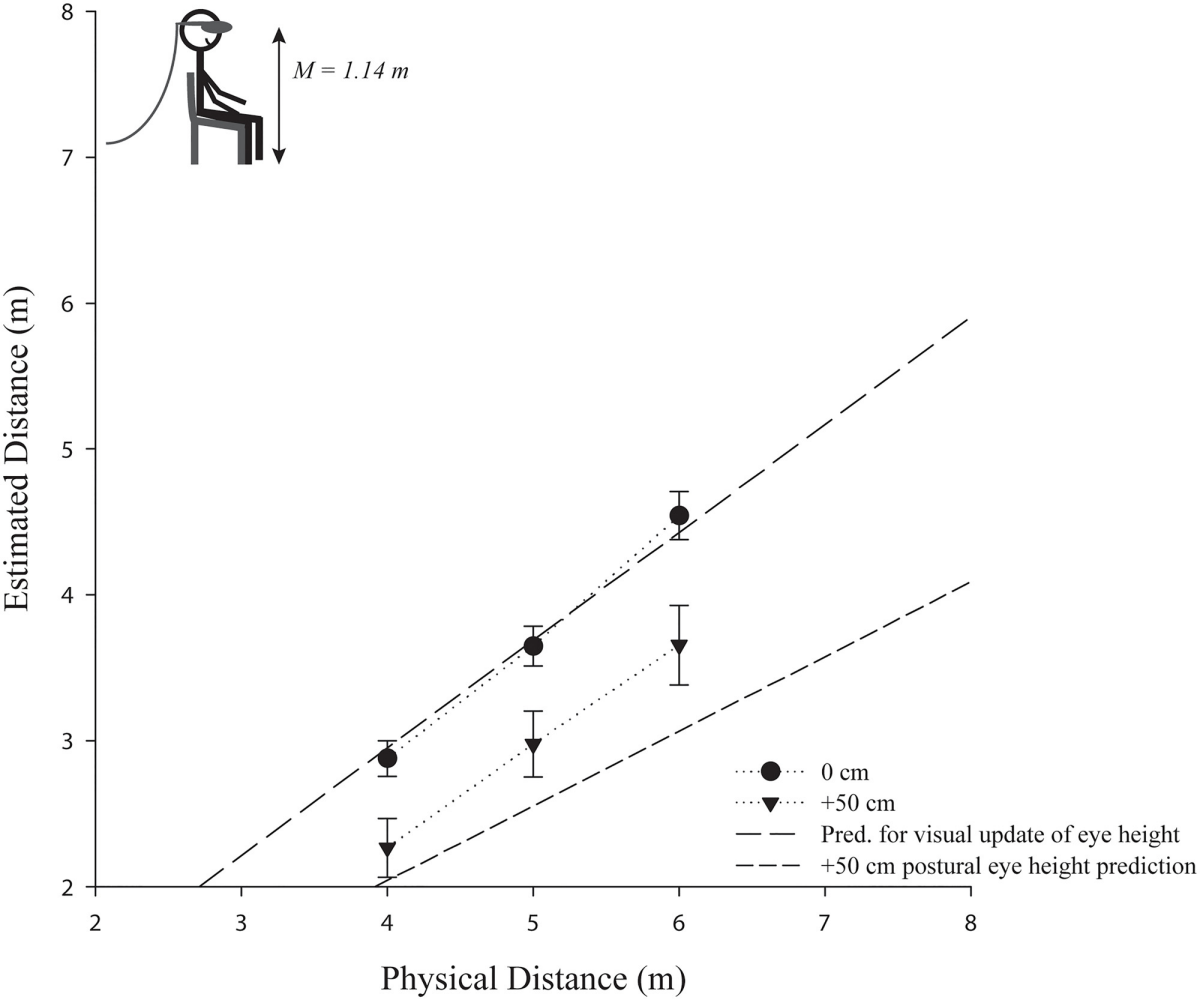
The effect of a manipulated virtual eye height (+50 cm) on egocentric distances in a sitting posture in comparison to the respective baseline condition (0 cm). Error bars represent ±1 SE. The actual mean participant (postural) eye height in the experiment is depicted in the left upper corner. **Note:** (a) The predictions are shifted by the observed underestimation in the baseline condition to account for the usually observed distance underestimation in head mounted displays (in an ideal world, the 0 cm estimates to veridical performance). (b) If the virtual eye height were used, there should be no differences and the prediction for visual eye height would apply for all conditions.

Supporting the hypothesis of using postural cues to determine eye height in VR, a repeated measures ANOVA confirmed that the distance estimates were significantly higher in the 0 cm eye height condition (*M* = 3.69, *SE* = 0.13) compared to the +50 cm condition (*M* = 2.97, *SE* = 0.23), *F*(1, 21) = 7.67, *p* = .012, *ŋ*_*p*_^2^ = .27 (see Fig 7). In addition, there was an interaction between eye height condition and distance, *F*(2, 42) = 3.34, *p* = .045, *ŋ*_*p*_^2^ = .14, with the differences between the eye height conditions increasing as a function of increase in distance, which is in line with the postural eye height hypothesis (see Fig 3). Furthermore, we also tested the converse prediction. If distance judgments would have been based on an internalized standing eye height, the +50 cm condition of Experiment 2 should yield similar estimates than those observed in the baseline condition (0 cm) of Experiment 1. However, an independent samples t-test confirmed, that they are reliably different, *t*(27) = -3.61, *p* < 0.01, supporting the idea that eye height is determined by postural cues according to the body posture.

## Experiment 3: Determining eye height in an uncommon lying posture

The results of Experiments 1 and 2 suggest that the visual information provided in the virtual environment seen through a HMD may not be dominant or reliable enough for determining eye height, if the potential sources of information are in conflict. However, these experiments do not fully resolve whether participants use an internalized eye height informed by experience of the posture or whether it is determined in real-time according to postural information. Standing and sitting are very common postures so experience in these postures and consequently, an internalized value could have informed eye height. To investigate whether experience is necessary to determine eye height in VR, we put participants in a less common posture: lying prone on a bed. If experience is important, we would expect participants to rely mostly on an internalized standing eye height. If eye height is determined by postural information in real-time, then the participants should use their postural information from getting on the bed to specify their new eye height with respect to the ground surface.

### Method

#### Participants

Forty-two paid (22 female) participants were recruited from the university community of Tübingen, Germany. All had normal or corrected to normal visual acuity and were screened for the ability to fuse stereo displays. The age ranged from 16 to 48 years (*M* = 27.33).

#### Stimuli and apparatus

**Fig 8:**
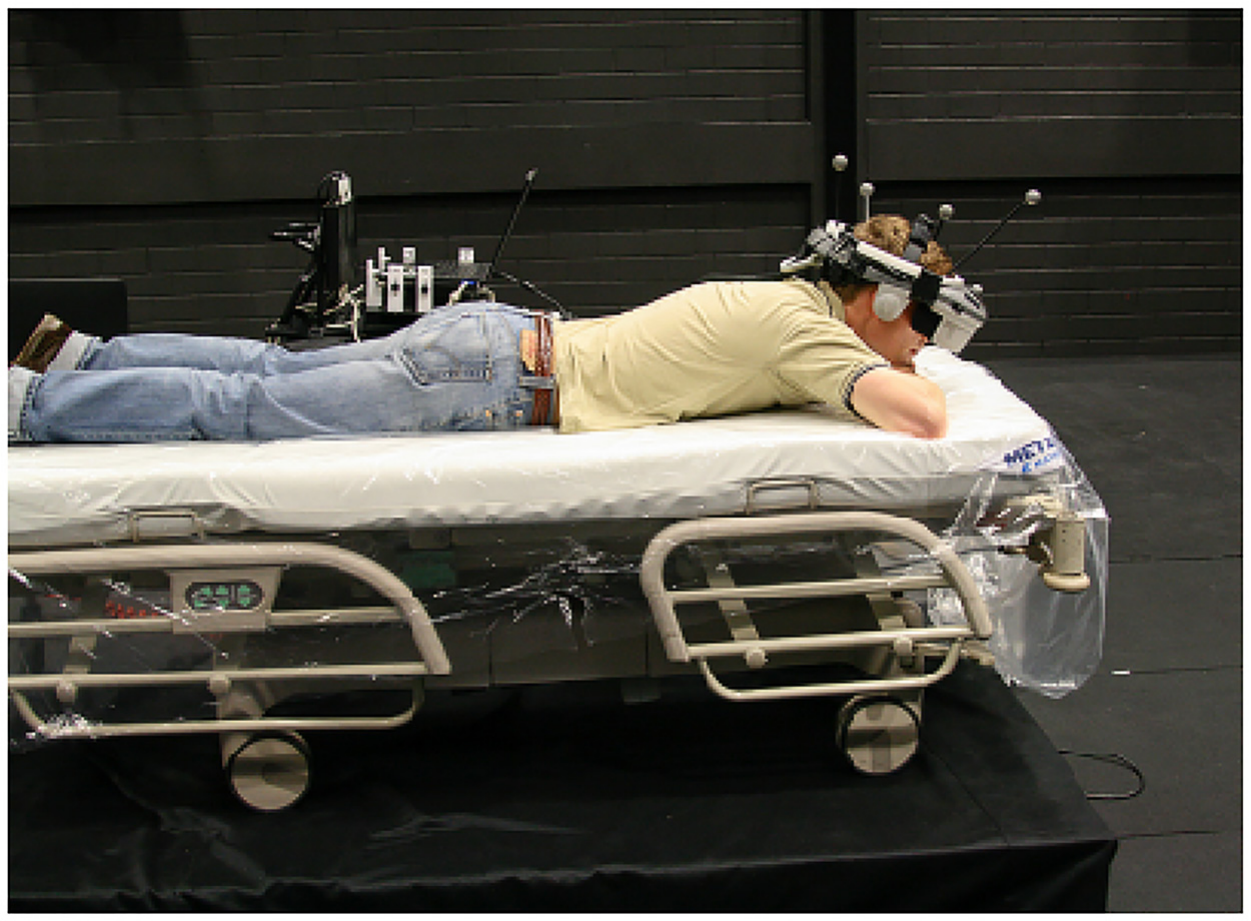
Participant judging distances in the sparse-cue virtual environment in a lying prone posture on an adjustable hospital bed. The individual in this image has given written informed consent (as outlined in PLOS consent form) to publish these case details.

For Experiment 3, we used the same technical setup and virtual environment as in Experiment 1. Participants completed the distance judgments while lying prone on an adjustable hospital bed (model Evolution MA 2, Hill-Rom, Batesville, IN, USA, see Fig 8).

### Design and procedure

Participants started in a different room than the one in which the experiment was conducted. The participants received written and verbal instructions. To allow for lowering and raising the virtual eye height, while still being able to climb on the bed directly from the floor, it was adjusted to reflect the approximate seated eye height of the participant (adjusted by the experimenter before the participant entered the room). The experimenter showed the participants the meter stick, until they indicated that they had a good image of the stick in mind. The experimenter then guided the participants into the tracking space and instructed them to get on the bed and repositioned them to ensure that all participants had approximately the same lying position on the bed. After donning the HMD, the experiment began with the same exploration phase as in Experiments 1 and 2. The participants were randomly assigned to only one of three conditions (between-subject design). The visually specified eye height either: (1) matched the actual eye height (0 cm), (2) was 50 cm lower than the actual eye height (-50 cm, here the position of the head could not go lower than the bed surface in comparison to Experiment 2), or (3) was 50 cm higher than the actual eye height (+50 cm). Participants completed 18 trials (4, 5 and 6 meters, each six times in a random blocked order). The procedure for reporting the distances was the same as in Experiments 1 and 2.

### Results

Due to technical errors with the HMD, the data of four participants were excluded from the analysis. We analyzed the verbal distance estimates using a repeated measures ANOVA with distance (4, 5, 6 m) and repetition (1–6) as within-subjects factors, virtual eye height (-50, 0, or +50 cm) as a between-subjects factor, and distance estimates as the dependent variable. As expected, distance was significant, with the estimates of distance increasing linearly with increasing distance, *F*(2, 70) = 201.51, *p* < .001, *ŋ*_*p*_^2^ = .85.

**Fig 9:**
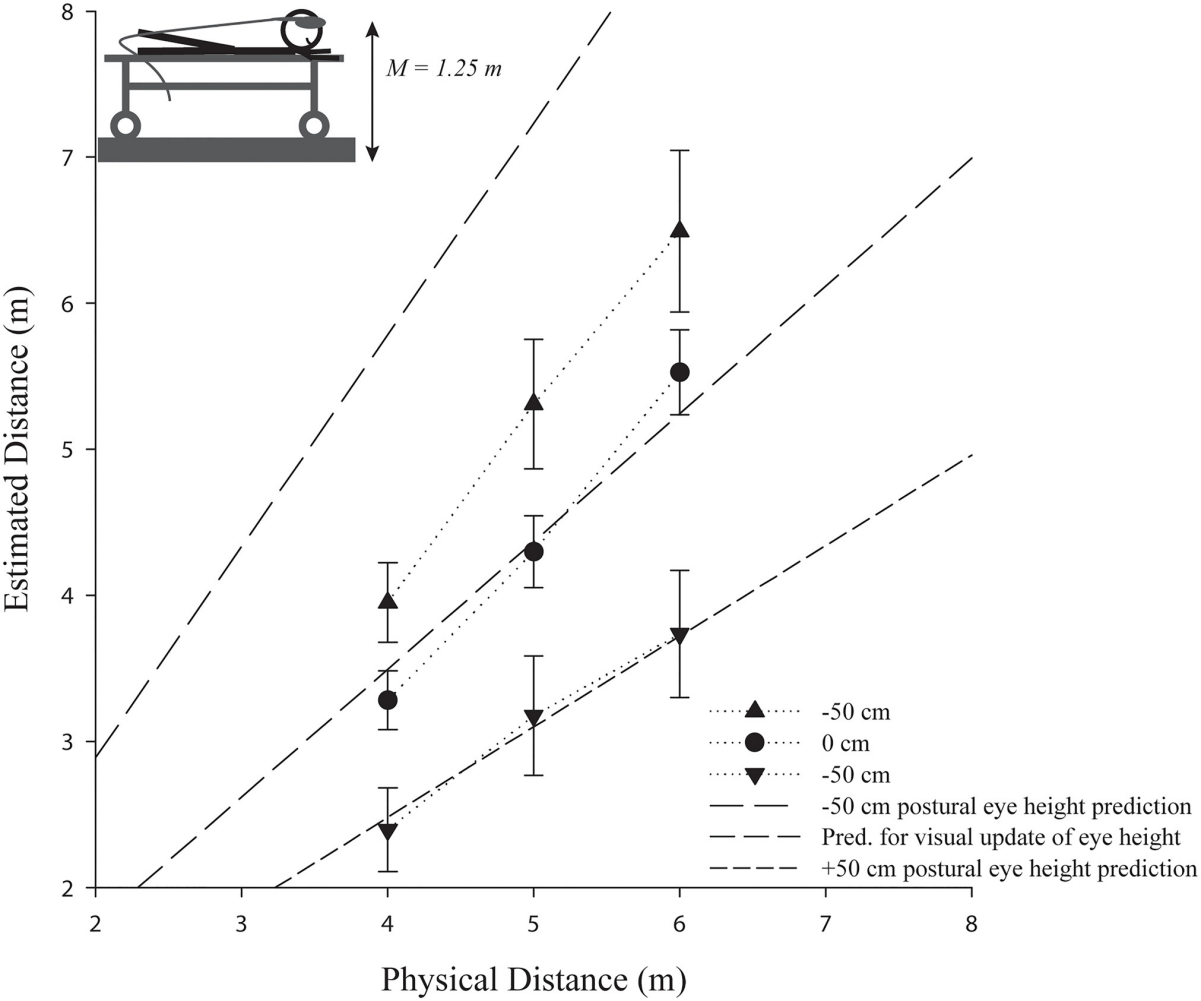
The effect of a manipulated virtual eye height (-50 cm or +50 cm) on egocentric distances in a prone position on a bed (adjusted to be approximately at seated eye height) in comparison to the respective baseline condition (0 cm). Error bars represent ±1 SE. The actual mean participant (postural) eye height in the experiment is depicted in the left upper corner. Note: (a) The predictions are shifted by the observed underestimation in the baseline condition to account for the usually observed distance underestimation in head mounted displays (in an ideal world, the 0 cm estimates would correspond to the prediction, which is veridical performance). (b) If the virtual eye height were used, there should be no differences and the prediction for visual eye height would apply for all conditions.

The repeated measures ANOVA revealed that the eye height manipulation had a significant effect on the estimated distances in the -50 cm (*M* = 5.25, *SE* = 0.41), 0 cm (*M* = 4.37, *SE* = 0.24), and +50 cm (*M* = 3.10, *SE* = 0.38) eye height conditions, *F*(2, 35) = 9.32, *p* = .001, *ŋ*_*p*_^2^ = .35. These results suggest that the participants did not rely on their visual information to determine their eye height to judge the egocentric distances (see Fig 9). Post hoc pairwise comparisons using Bonferroni correction confirmed significant differences between the -50 and 0 cm eye height conditions, *p* = .047, and the -50 and +50 cm conditions, *p* < .001. However, there was no reliable difference between the +50 and 0 cm conditions, *p* = .241, which may have been due to the greater variability of estimates in the prone position. In addition, the predictions for the 0 cm and +50 cm conditions differ by a smaller amount than the predictions for the -50 cm and 0 cm conditions. As in the previous experiments, there was an interaction between eye height condition and distance, *F*(4, 140) = 6.47, *p* < .001, *ŋ*_*p*_^2^ = .27, with the differences between the eye height conditions increasing as a function of distance, supporting the postural eye height hypothesis.

In contrast to the postures used in Experiments 1 and 2, there were two different eye-height possibilities for the prone position. The participants could use their physical standing eye height (i.e., distance from their eyes to their feet), because the lack of experience in such a posture, which would suggest the use of an internalized eye height (c.f. [13]) when uncertain about the posture. The other predictor is the actual distance from the participants’ eyes to the floor while lying prone, suggesting the use of postural information to ensure the necessary flexibility of perceived eye height to motor experiences. To investigate which eye height was used, we calculated (cf. equation Fig 3) the distances based on our predictors of their standing and actual eye height (mean eye height during the experiment—acquired from the motion capture data from tracking the HMD). We found that actual postural eye height had the best fit (linear regression with backward elimination), *β* = .53, *t*(36) = 3.69, *p* = .001. The predicted distances based on the model of eye height informed by postural cues also explained a significant proportion of variance in the participants’ distance estimates, *R* ^2^ = .28, *F*(1, 36) = 13.64, *p* = .001. These results support the hypothesis that eye height in VR is informed constantly by postural cues across changes in body posture and consequently, does likely not depend on an internalized value for eye height.

## Experiment 4: The reliability of visual information for determining eye height in virtual environments

The results of Experiments 1–3 suggest that our participants relied more on their postural cues informed in real-time for determining their eye height in VR, and not on what they visually perceived. Nevertheless, it is valuable to investigate whether a lack of visual information was the reason that participants relied more on the postural information in the virtual environment. For example, missing visual cues such as familiar size cues, which are usually available in a rich-cue environment, or the incomplete stereo information due to the optics of the HMD and an accommodation-convergence mismatch might be reasons why postural information is so important for determining eye height in VR with our setup. Thus, we conducted another experiment where participants were required to rely on the visual information provided within two virtual environments (sparse and rich-cue) seen in the same HMD and were asked to directly estimate their virtual eye height in an adjustment task. If the visual information present in the virtual environments is sufficient to determine eye height, participants should be able to quite accurately adjust the virtual camera to match their actual eye height.

### Method

#### Participants

Twenty-five paid (12 female) participants were recruited from the university community Tübingen, Germany. All had normal or corrected to normal visual acuity and were able to fuse stereo images. Age ranged from 21 to 47 years (*M* = 28.0).

#### Stimuli and apparatus

We used the same technical setup and the same virtual environment (sparse-cue) as in Experiment 1–3 along with a second environment (rich-cue), which was a replica of a real office and provided a wealth of familiar size cues (chairs, tables, doors, etc.). To control the virtual eye height, participants used a gamepad to adjust the position of the camera in the y-axis.

#### Design and procedure

All participants received written and verbal instructions and then the experimenter guided the participants to their standing position and helped them to don the HMD. Each environment included six training trials to familiarize the participants with the adjustment task where the participant adjusted the virtual camera’s height to match their physical eye height. The camera either started 50 cm above or below their physical eye height in counterbalanced order. After four training trials, each participant completed 24 adjustment trials. When the participant finished one environment, the procedure was repeated with the second environment (counterbalanced, 48 trials in total).

### Results

Adjustment trials were transformed into ratios by dividing the adjusted eye height by the real eye height of the corresponding participant. This means, a ratio of 1.0 reflects a perfect match between the visual virtual eye height and the real physical eye height of the participant. One participant was removed from the analysis for being more than 3 *SD* above the mean.

We analyzed the eye height ratios using a repeated measures analysis of variance (ANOVA) with environment (sparse-cue and rich-cue) and repetition (24) as within-subjects factors and order as a between-subjects variable, and the ratios as the dependent measure. There was no effect of order for the ratios, *p* = .79.

**Fig 10:**
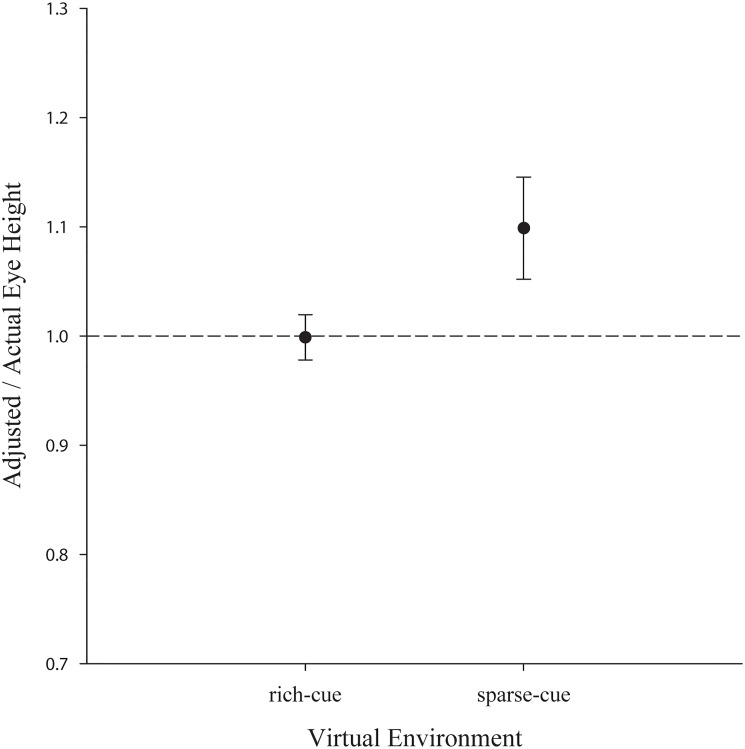
Mean ratio adjusted/actual eye height for the tested environments. Error bars represent ±1 SE.

There was only a marginal effect of the environment, with the averaged ratios in the sparse-cue environment being overestimated (*M* = 1.12, *SE* = 0.05, standard errors denote between-subject errors) compared to the rich-cue environment (*M* = 1.01, *SE* = 0.02), *p* = .064. This suggests that if we consider the group average, minimal visual information might already be sufficient to determine eye height (see Fig 10).

In the rich-cue environment, the participants were veridical on average, and in the sparse environment, they were close to veridical in visually adjusting their virtual eye height to their corresponding physical eye height, albeit overestimated. These results suggest that, on average, eye height could be approximately determined based on the available visual information in our experiments, suggesting that our obtained results might not be due to a complete lack of visual information for perceived eye height. Nevertheless, we observed quite some between-subjects variability in the adjustment task with a greater variability in the environment, where no cues except HMD stereo and linear perspective were present. In fact, when considering the absolute mean error in the visual eye height estimates in the rich-cue (*M* = 13.24 cm, *SE* = 2.09 cm) and sparse-cue (*M* = 34.04 cm, *SE* = 5.53 cm) environments, visually specified eye height might not be as veridical as the average results indicate. These results suggest that there is individual variability with regard to tendency to overestimate or underestimate visually adjusted eye height leading to a close to veridical average performance.

However, examples from the real world show that judging visually perceived eye height seems to be quite variable. For example, it has been shown that participants slightly underestimate their standing eye height, and misjudge their eye height with respect to a lower ground plane when standing on an elevated ground plane of two meters height (see [13]). Related to eye height, real world experiments investigating for instance the perceived eye level have shown that if humans judge their eye level, variability amounts to ~1° visual angle, and the judgments are off by 2.2° of visual angle (e.g. [5]), even when the head is fixed and stable. In this specific task, the absolute error corresponds to 13.9 cm underestimation when judging eye level over a distance of 2.4 meters (c.f. [5]). Thus, we can tentatively conclude that although individuals’ estimates of their eye height in VEs were variable, the amount of this variability is very similar to the variability reported in similar domains (e.g. also eye level) for other measures conducted in the real world. Indeed, this variability in individual assessments of visually perceived eye height in both real and virtual environments may provide a motivation for the perceptual system to take postural information about eye height into account.

## Experiment 5: Visually determining different virtual eye heights within virtual environments

The results of Experiment 4 suggest that our participants were on average able to adjust the virtual camera to their physical/postural eye height. However, the judgments were quite variable, which could be an indication why the participants relied more on postural information to determine their eye height for perceiving distances in virtual environments. We further wanted to investigate how accurately different visual eye heights can be estimated with the visual information available within virtual environments. In the previous experiment, our participants were required to adjust the camera in the virtual scenes so that it matches their physical eye height. In this study, we instead asked participants to verbally judge several different virtual eye heights. If the visual information in the virtual environment was not reliable or insufficient, we would expect that the judgments would be inaccurate and highly variable.

### Method

#### Participants

Twenty-two paid (10 female) participants were recruited from the university community Tübingen, Germany. All had normal or corrected to normal visual acuity and were able to fuse stereo images. The age of the participants ranged from 21 to 55 years (*M* = 32.59).

#### Stimuli and apparatus

We used the same technical setup (except the game pad) and the same virtual environments (sparse-cue and rich-cue) as described in Experiment 4.

#### Design and procedure

All participants received written and verbal instructions and were shown a meter stick with additional labels every 10 centimeters until they indicated that they had a good representation of the length of the stick. Then, the experimenter guided the participants to their standing position and helped them to don the HMD. In each environment, the participants started the experiment by verbally judging three different virtual eye heights (rich-cue: 0.8 m, 1.6 m and 3.2 m; sparse-cue: 0.6 m, 1.8 m, 3.6 m) in a randomized order to familiarize the participants with the task. These trials were discarded for analysis and the virtual eye heights presented in these training trials were not used in the actual judgment trials. After these training trials, the participants were required to judge varying virtual eye heights (0.5 meters to 4.0 meters in 25 centimeter steps, each virtual eye height was repeated two times in a fully randomized order). When the participant completed thirty trials (excluding training trials), the procedure was repeated in the second environment (within-participant design). The order in which the environments were presented was counterbalanced between participants.

### Results

Three participants were removed from the analysis for being more than 3 *SD* above or below the mean virtual eye height estimates in the sparse-cue environment as is typical in designs using verbal estimates. We analyzed the verbal eye height estimates using a repeated measures ANOVA with environment (rich-cue and sparse-cue), virtual eye heights (0.5 m to 4.0 m in 25 cm steps) and repetition (two) as within-subjects factors, order (rich or sparse-cue first) as a between-subjects factor, and the verbal virtual eye height estimates as the dependent measure. As expected, the virtual eye height was significant, with the estimates of the virtual eye height increasing linearly from the 0.5 m to the 4.0 m eye heights, *F*(14, 238) = 122.87, *p* < .001, *ŋ*_*p*_^2^ = .88.

**Fig 11:**
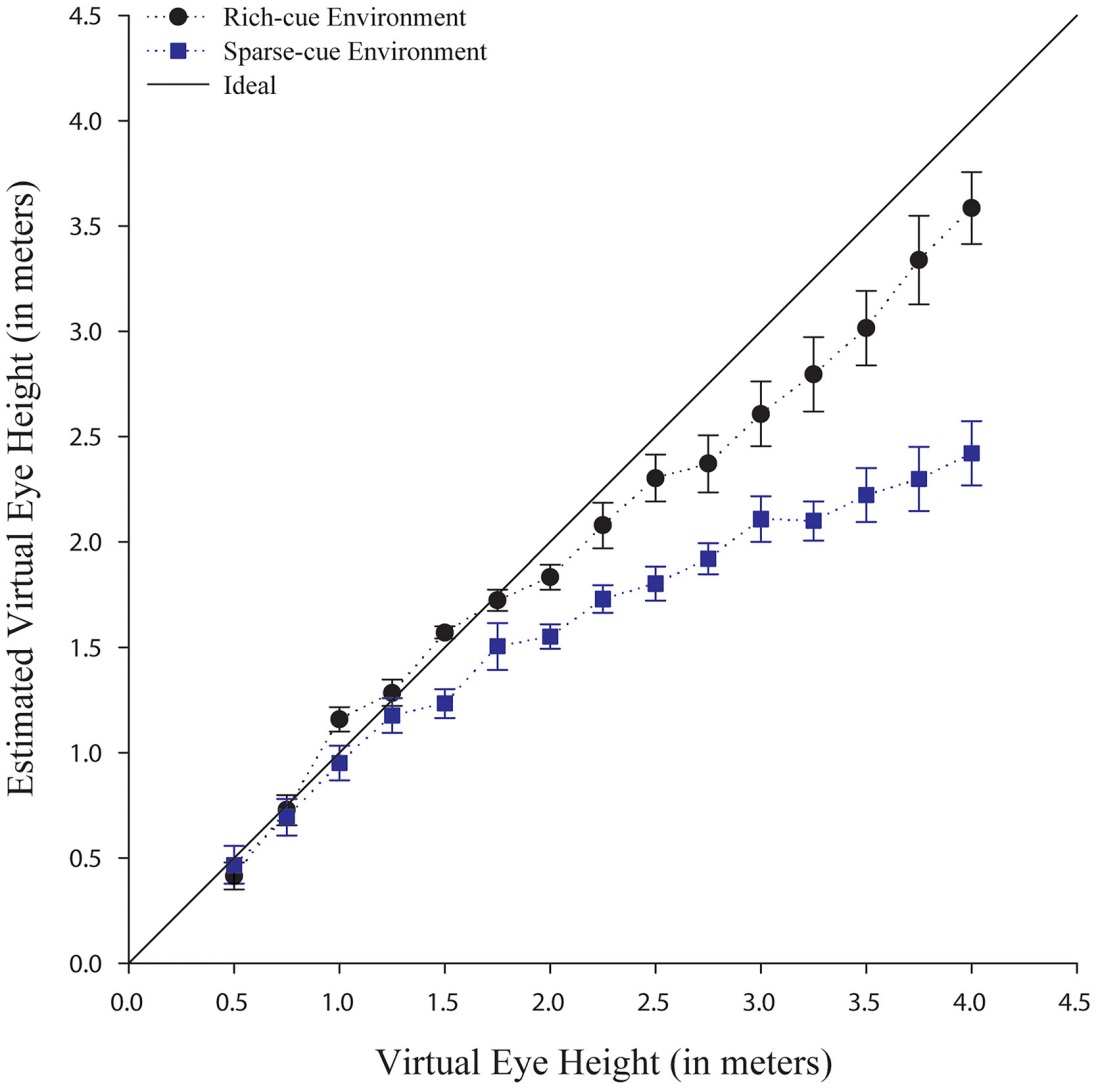
Verbal estimates of different virtual eye heights in the rich-cue (black circles) and sparse-cue (blue squares) environment. Error bars represent ±1 SE.

The repeated measures ANOVA also revealed that the virtual eye height judgments varied depending on the environment. The verbal judgments were significantly lower (actual mean of all tested virtual eye heights is 2.25 meters) in the sparse-cue environment (*M* = 1.61, *SE* = 0.07) compared to the rich-cue environment (*M* = 2.05, *SE* = 0.09), *F*(1, 17) = 30.73, *p* < .001, *ŋ*_*p*_^2^ = .64 (see Fig 11). The judgments of the virtual eye heights did not vary as a function of the order in which the environments were presented to our participants, *p* = .177.

In order to assess the degree of errors, we transformed the verbal estimates into ratios by dividing the estimated eye height by the actual virtual eye height. We conducted repeated measures ANOVA with the virtual eye height and environment as independent variables and the ratios as the dependent measure. Environment was significant with eye heights in the sparse cue environment (*M* = 0.77, *SE* = 0.04), being more underestimated than those in the rich cue environment, (*M* = 0.94, *SE* = 0.03), *F*(1, 18) = 14.14, *p<*.001, *ŋ*_*p*_^2^ = .44. Eye height was also significant with underestimations increasing as a function of increasing eye height, *F*(14, 252) = 4.56, *p* < .001, *ŋ*_*p*_^2^ = .20 (see Fig 11). The estimates do not drastically differ from 1.0 (i.e. perfect accuracy) until after about 2 m, where participants begin to err more than 10%.

Considering the single estimates for the differing virtual eye heights and the inherent variability in our measure (verbal estimates), our participants were able to quite accurately judge the differing eye heights, at least up to about ≤ 2 meters. In both environments, the participants underestimated the virtual eye heights that were higher than 2 meters and this underestimation increased with increasing distance to the virtual ground plane. Importantly, in both environments, the verbal judgments are quite accurate and less variable for the manipulated eye heights used in Experiments 1–4 (i.e. 0.5 meters out to 2 meters), but were underestimated for larger eye heights. However, for the virtual eye heights above 2 meters, the average absolute error substantially increases, especially in the sparse-cue environment, where no additional cues except stereo, linear perspective and texture density were available. In both environments, the rich and sparse-cue environments, the errors rapidly increased, suggesting that estimates of virtual eye heights greater than 2 meters are quite compressed. More work should be conducted to investigate the origin of this compression, although, it is beyond the scope of the current research questions. Consistent absolute errors were also observed in the real world when using a perceptual matching task to judge eye height visually to a ground plane with eye heights ranging from approximately 3.25 meters to 3.75 meters, see [13].

In summary, the results of Experiment 5 suggest, that at least some visual information is usable to visually determine the virtual eye height if little possibility for calibration to the virtual space is provided, given that these eye heights are within a given range below 2 meters. The manipulated eye heights in Experiments 1–3 were on average in the range of a minimum of 0.75 meters to a maximum of 2.08 meters. Thus, according to the results of Experiment 5, eye height can be reliably and somewhat accurately determined by visual information provided it is within this range. Even so, at least in virtual environments, people may experience the information as unreliable (depending on their immersion), which could also be a reason why humans might rely more on postural cues to determine their eye height, especially when little possibility for calibration is provided. Whether this also applies to a real world scenario is an open question.

## General Discussion

Prior to our experiments, the sensory modality potentially used to specify eye height in virtual environments had not yet been empirically investigated for tasks in action space. In this set of experiments, we found that there are instances where humans seem to rely more on their postural information for determining eye height in virtual environments as demonstrated by the predictable variations in their distance estimates. Our Experiments 1–3 demonstrate that variations in the virtual eye height have predictable effects on perceived egocentric distances and that these effects are consistent across different postures. This indicates that determining eye height in VR is likely achieved by relying on postural cues informing eye height in real-time, and is consequently not dependent on an internalized representation of eye height (c.f. [13]). Thus, these results suggest, that in some contexts, individuals may rely more on postural cues to determine eye height for distance perception in VR, *even though* virtual environments mainly stimulate the visual modality. Nevertheless, the results of our Experiments 4–5 suggest that our observed effects on perceived distances due to a reliance on postural cues is not the result of a complete absence of visual cues to eye height within the virtual environment, although visually perceived eye height in VR seems to be quite variable. However, the results of Experiment 5 suggest that the visual information even in a sparse-cue environment only providing stereo, density and linear perspective cues is sufficient to estimate virtual eye heights quite accurately in near space. Thus, we argue that our observed effects are likely not the result of an absence of visual cues to virtual eye height, but likely the result of a diminished reliability of visual cues to eye height. This is also suggested by the variability in the visual judgments of virtual eye height in our Experiments 4–5 and comparable experiments in the real world (e.g. [5, 13]). Indeed, this variability in visually determining eye height in VR might provide an incentive for the perceptual system to take postural information about eye height into account (c.f. [11]).

Our results are in line with a large body of literature, suggesting that eye height is very important in the context of visual perception (e.g. [1, 2, 3, 4, 7, 11, 12]), especially for perceiving egocentric distances (e.g. [5, 6, 16]). Specifically, these results are in line with and support other findings, highlighting the importance of postural cues in visual perception not only within virtual (e.g. [20, 21, 22]), but also real environments [3, 7, 11, 12]. They also demonstrate that in an environment with a regular ground surface [23] the body may contribute important information for a visual task, like egocentric distance perception (e.g. [7, 19, 20]). These findings are consistent with and extend previous literature stating that eye height is an important source of information for perceiving different aspects of the spatial layout of our environment (e.g. [1, 2, 24]). Most importantly, the present work reveals that even in a task, which does not involve locomotion [20, 21] or navigation [22] postural cues can be important for the perception of the spatial layout of our surrounding (virtual) environment.

However, how our findings generalize to other environments (e.g. real world scenarios [8, 10, 11, 12, 13]) and to different tasks (e.g. perceptual-motor tasks, where one can calibrate to the visual environment by gaining feedback, see e.g. [8, 10, 14, 15, 25]), is an open question. Our results are in line with research conducted in the real world, which shows that eye height is accessible even if no perceptual-locomotor or other specific calibration feedback is provided [1, 2, 12]. For example, manipulating eye height using a false or elevated floor influences perceived size when no direct perceptual-motor feedback was provided (see [1, 2]). However, related work also suggests that providing perceptual motor feedback in action space can provide the opportunity to interpret the available visual information with respect to one’s own movements, thereby resulting in accurate judgments of distance in virtual environments (see for example [25, 26]). Hence, it is possible that additional information provided via perceptual-motor calibration is a main source of information specifying eye height, and that postural information is mainly used in the absence of this information. In support of this notion, recent research in near space suggests that a perceptual-motor calibration of eye height to a new support surface (e.g. a table) is necessary for accurate reaching performance (see e.g. [8, 10]). Thus, other paradigms using an experimental apparatus to elevate or lower participants without them noticing this motion (i.e. moving them sub-threshold, see [27]) could be used to achieve a greater control over the possible sensory inputs for perceiving eye height (i.e. visual, vestibular, body posture from proprioception). Furthermore, with such a setup it might be possible to manipulate all of those inputs in a single paradigm to investigate this question further.

Future research should be conducted to fully understand when and why postural cues are used to determine eye height, not only in virtual environments but also in real environments and whether eye height in VR is still determined by postural cues when perceptual-motor calibration is possible (as in [8, 10]). In addition, further studies are required to fully disentangle what we describe as postural cues, which likely include but are not limited to proprioceptive, haptic and/or vestibular cues (see [9]). Such research is needed, because one explanation for our experimental results is that all available cues for determining eye height from various modalities, including vision, are integrated along a Bayesian multi-sensory integration approach, where cues are weighted with respect to their reliability across the various contexts (e.g. see [23] for such an approach). In our experimental context, it could be that cues to eye height from other modalities were weighted higher than the visual cues potentially specifying eye height; thereby leading to the observed influences on perceived distance. Nevertheless, our results also indicate that while eye height seems to be mainly informed by postural cues, it is combined with the angle of declination, which is thought to be mainly informed by vision (e.g. [5]). In our experiments, both sources of information from different modalities seem to be consistently used in combination across different postures to ensure perceptual constancy (in contrast to other theories, see [28]). Such a mechanism would allow individuals to perceive an unchanging surrounding environment.

It is also important to further explore whether limitations in the VR technology are a reason for our observed reliance on postural cues for determining eye height in virtual environments instead of visual information. Specifically, the accommodation-convergence mismatch in a HMD (see [14, 29]) could be a reason for the quite variable judgments of visually perceived eye height in our virtual environments and consequently the cause that our participants relied more on their postural cues for determining eye height. However, it seems unlikely that this limitation is a main factor for the observed reliance on postural cue. While there is compelling evidence that an accommodation-convergence mismatch has effects on perceived distance (at least in reaching space, see [14, 29]), it is doubtful that this fully accounts for the strength of our observed effects in action space, especially if we consider the results of Experiment 5. Specifically, the effect of such a mismatch is usually that convergence is pulled towards the accommodation distance (c.f. [14]). The HMD used in our experiments features collimated optics, which simulate an accommodation distance at infinity. Thus, convergence should be pulled towards infinity, which should result in an overestimation of distance (and the visually perceived virtual eye height) as observed for reaching distances (see [14]). Furthermore, all conditions should be similarly affected by such a mismatch, because if perceived distance was affected by, for example, stereo information, it should be overestimated by a similar amount across all conditions, still preserving any differences between conditions. Thus, the observed reliance on postural cues to determine eye height might even be a general phenomenon rather than specific to virtual environments in the event that little calibration to an altered visual environment is provided. Therefore, more research in both virtual and real worlds are needed.

Finally, our research results are not only important for perception researchers, but also have implications users and developers of many VR setups and applications emphasizing the importance of our results for everyone working with VR technology. Considering the simplicity of achieving a conflict between visual and postural information specifying eye height in virtual environments (either on purpose like in our studies or by accident), it is extremely important for VR application designers to consider the user’s posture and eye height to maintain the perceptual fidelity of the virtual environments. According to our results, every discrepancy between the different sensory modalities potentially used for determining eye height in VR will likely alter the perceived virtual space in VR setups, where due to technical, space or time constraints only little calibration to the virtual environment is possible. This applies to many VR applications and scenarios existing today. For example, consider a multi-user scenario where successful collaboration between the users is hindered, because only one user might have a correct perspective and virtual eye height. According to our results the users will likely perceive the virtual space differently. However, our results also indicate that because of the reliance on postural cues in virtual environments, purposeful manipulations of the virtual eye height could also be a solution to alter the perceived space in VR in desired ways [30].

## References

[1] Wraga M. The role of eye height in perceiving affordances and object dimensions. Perception & Psychophysics 1999; 61 (3): 490–507. 10334096 10.3758/bf03211968

[2] Wraga M. Using Eye Height in Different Postures to Scale the Heights of Objects. Journal of Experimental Psychology: Human Perception and Performance. 1999; 25 (2): 518–530. 10205864 10.1037//0096-1523.25.2.518

[3] Lee DN, Kalmus H. The Optic Flow Field: The Foundation of Vision [and Discussion]. Philosophical Transactions of the Royal Society B: Biological Sciences. 1980; 290 (1038): 169–179. 10.1098/rstb.1980.0089 6106236

[4] Gibson JJ. The ecological approach to visual perception. Boston, MA, US: Houghton, Mifflin and Company 1979 xiv, 332- p.

[5] Ooi TL, Wu B, He ZJ. Distance Determination by the Angular Declination Below the Horizon. Nature. 2001; 414: 197–200. 11700556 10.1038/35102562

[6] Sedgwick HA. The visible horizon: A potential source of visual information for the perception of size and distance. US 1973; 1301–1302 p.

[7] Proffitt DR, Linkenauger SA. Perception viewed as a phenotypic expression. Action Science: Foundations of an Emerging Discipline. 2013; 171.

[8] Coats RO, Pan JS, Bingham GP. Perturbation of perceptual units reveals dominance hierarchy in cross calibration. Journal of Experimental Psychology: Human Perception and Performance. 2014; Vol 40 (1): 328. 10.1037/a0033802 23895390

[9] Mittelstaedt H. Origin and processing of postural information. Neuroscience & Biobehavioral Reviews. 1998; 22(4), 473–478. 9595557 10.1016/s0149-7634(97)00032-8

[10] Pan JS, Coats RO, Bingham GP. Calibration is action specific but perturbation of perceptual units is not. Journal of Experimental Psychology: Human Perception and Performance. 2014; 40 (1): 404 10.1037/a0033795 23937217

[11] Warren JWH, Whang S. Visual Guidance of Walking Through Apertures: Body Scaled Information for Affordances. Journal of Experimental Psychology: Human Perception and Performance. 1987; 13: 371–383. 2958586 10.1037//0096-1523.13.3.371

[12] Mark LS. Eyeheight-Scaled Information About Affordances: A Study of Sitting and Stair Climbing. Journal of Experimental Psychology: Human Perception and Performance. 1987; 13 (3): 361–370. 2958585 10.1037//0096-1523.13.3.361

[13] Sinai MJ, Ooi TL, He ZJ. Terrain influences the accurate judgement of distance. Nature. 1998; 395 (6701): 497–500. 9774104 10.1038/26747

[14] Bingham GP, Bradley A, Bailey M, Vinner R. Accommodation, occlusion, and disparity matching are used to guide reaching: A comparison of actual versus virtual environments. Journal of Experimental Psychology: Human Perception and Performance. 2001; 27 (6): 1314 11766927 10.1037//0096-1523.27.6.1314

[15] Waller D, Richardson AR. Correcting distance estimates by interacting with immersive virtual environments: Effects of task and available sensory information. Journal of Experimental Psychology: Applied. 2008; 14 (1): 61–72. 10.1037/1076-898X.14.1.61 18377167

[16] Sedgwick HA. Section IV: Space and Motion Perception In: Boff K, Kaufman L, Thomas J, editors. Handbook of Perception and Human Performance. New York: Wiley-Interscience 1986.

[17] Glennerster A, Tcheang L, Gilson SJ, Fitzgibbon AW, Parker AJ. Humans ignore motion and stereo cues in favor of a fictional stable world. Current Biology. 2006; 16(4), 428–432. 16488879 10.1016/j.cub.2006.01.019 PMC2833396

[18] Loomis JM, Knapp JM. Visual Perception of Egocentric Distance in Real and Virtual Environments. Virtual & Adaptive Environments: Applications, Implications, and Human Performance Issues: 2003; 21.

[19] Leyrer M, Linkenauger SA, Bülthoff HH, Kloos U, Mohler B. The influence of eye height and avatars on egocentric distance estimates in immersive virtual environments. In Proc. Symposium on Applied perception in Graphics and Visualization. 2011; pp. 67–74.

[20] Campos JL, Byrne P, Sun H. The brain weights body-based cues higher than vision when estimating walked distances. European Journal of Neuroscience. 2010; 31 (10): 1889–1898. 10.1111/j.1460-9568.2010.07212.x 20584194

[21] Campos JL, Butler JS, Bülthoff HH. Multisensory integration in the estimation of walked distances. Exp Brain Res. 2012; 218 (4): 551–565. 10.1007/s00221-012-3048-1 22411581

[22] Waller D, Loomis JM, Haun DBM. Body-based senses enhance knowledge of directions in large-scale environments. Psychonomic Bulletin & Review. 2004; 11 (1): 157–163. 15117002 10.3758/bf03206476

[23] Wu B, Ooi TL, He ZJ. Perceiving Distance Accurately by a Directional Process of Integrating Ground Information. Nature. 2004; 428: 73–77. 14999282 10.1038/nature02350

[24] Dixon MW, Wraga M, Proffitt DR, Williams GC. Eye Height Scaling of Absolute Size in Immersive and Nonimmersive Displays. Journal of Experimental Psychology: Human Perception and Performance. 2000; 26 (2): 582–593. 10811164 10.1037//0096-1523.26.2.582

[25] Mohler BJ. The Effect of Feedback Within a Virtual Environment on Human Distance Perception and Adaptation. Dissertation at the University of Utah. ProQuest. 2007.

[26] Richardson AR, Waller D. Interaction with an immersive virtual environment corrects users’ distance estimates. Human Factors: The Journal of the Human Factors and Ergonomics Society. 2007; 49 (3): 507–517. 10.1518/001872007X200139 17552313

[27] Nesti A, Barnett-Cowan M, MacNeilage PR, Bülthoff HH. Human sensitivity to vertical self-motion. Experimental Brain Research. 2014; 232(1), 303–314. 10.1007/s00221-013-3741-8 24158607 PMC3898153

[28] Pylyshyn Z. Is vision continuous with cognition? The case for cognitive impenetrability of visual perception. Behavioral and Brain Sciences. 1999; 22 (3): 341–365. 11301517 10.1017/s0140525x99002022

[29] Hoffman DM, Girshick AR, Akeley K, Banks MS. Vergence-accommodation conflicts hinder visual performance and cause visual fatigue. Journal of Vision. 2008; 8 (3): 33 10.1167/8.3.33 18484839 PMC2879326

[30] D'Cruz M, Patel H, Lewis L, Cobb S, Bues M, Stefani O, et al Demonstration: VR-HYPERSPACE—The innovative use of virtual reality to increase comfort by changing the perception of self and space IEEE Virtual Reality, IEEE, Piscataway, NJ, USA, 2014; 167–168.

